# The association of parents’ behaviors related to salt with 24 h urinary sodium excretion of their children: A Spanish cross-sectional study

**DOI:** 10.1371/journal.pone.0227035

**Published:** 2019-12-27

**Authors:** Esther Cuadrado-Soto, África Peral-Suarez, Elena Rodríguez-Rodríguez, Aránzazu Aparicio, Pedro Andrés, Rosa M. Ortega, Ana M. López-Sobaler

**Affiliations:** 1 Department of Nutrition and Food Science, Faculty of Pharmacy, Complutense University of Madrid, Plaza Ramón y Cajal S/N, Madrid, Spain; 2 Department of Chemistry in Pharmaceutical Sciences, Analytical Chemistry, Faculty of Pharmacy, Complutense University of Madrid, Madrid, Spain; 3 UCM Research Group: VALORNUT-920030, Department of Nutrition and Food Science, Faculty of Pharmacy, Complutense University of Madrid, Plaza Ramón y Cajal S/N, Madrid, Spain; Yenepoya Medical College, Yenepoya University, INDIA

## Abstract

**Background:**

Sodium intake is excessive among Spanish children, but the salt use behaviors of parents and children are unknown. This study aims to determine behaviors related to salt intake in both schoolchildren and parents and the relationship between parental behaviors and 24 h urinary sodium excretion (UNa-24h) in children.

**Subjects and methods:**

A convenience sample was taken from a cross-sectional analysis. Parents completed a self-reported questionnaire about their behaviors related to salt, and their responses were compared with the UNa-24h of their own children. The median test was used to identify differences in UNa-24h according to behaviors. Logistic regression was used to assess the relationship between the behaviors of parents and high sodium excretion in the children and the risk of children’s use of table salt, adjusting for age, sex, and BMI. Multinomial logistic regression models, adjusted by the covariates, were used to study the children’s salt preferences.

**Results:**

A total of 329 schoolchildren from different Spanish provinces were included in the study (mean age: 9.0 ± 1.2 years, 157 girls). The majority of families (parents mean age: 42.0 ± 5.2 years) reported adding salt to food during cooking (92%), and 59% of them never looked at the sodium content on food labels. However, none of these behaviors were related to UNa-24h (p > 0.05). The use of iodized salt (53%), the presence of a salt shaker on the table (6%), and the use of table salt by fathers (57%), mothers (52%) or children (17%) increased the odds (p < 0.05) of children having a higher UNa-24h. Checking sodium content on food labels and the use of table salt by the children or father was associated with a lower preference for salty foods (p < 0.05).

**Conclusions:**

It is important to make parents aware of the relationship between their behaviors regarding the use of discretionary salt and their children's sodium intake. Our data suggest that salt-specific education programs on how to reduce salt both in-home and outside the home should be implemented to improve behavior skills related to salt consumption in parents and children.

## Introduction

Cardiovascular diseases (CVDs) are the leading cause of death in Europe, with more than 3.9 million deaths per year due to CVD [1]. Excess sodium intake is linked to raised blood pressure, which is a major risk factor for cardiovascular-related conditions such as stroke and heart attacks [2]. In addition, it has been suggested blood pressure tracks with age [3,4], and for this reason, there is a need to reduce the dietary risk factors such as sodium consumption associated with high blood pressure in children [3]. The adoption of a healthy diet at an early age is crucial for the prevention of CVD [5].

In Spain, nationally representative data shows that the population exceeded sodium intake [6–8]. Partearroyo et al. have recently studied sodium intake in the Spanish ANIBES population, with adults and children aged 9–75 years (n = 2009). The authors reported a dietary sodium intake of 2025 ± 805 mg/day, using a 3-day dietary record and excluding salt added at table or during cooking [6]. Although there is extensive scientific evidence regarding the consumption of excessive sodium in various populations, there are fewer data available for children than for adults [9]. In a recently conducted analysis by Aparicio et al. [10], the researchers found that, on average, Spanish children aged 7–11 years excreted 3052 ± 1182 mg/day of sodium. According to WHO criteria, the results of this study imply that 84% of the subjects who were under 10 years old consumed >4 g of salt per day and that 67% of those aged >10 years had an intake of >5 g salt/day [11].

Dietary behaviors, including the choice of foods that are high in sodium content, are established during childhood and track over time [12,13]. However, it has been observed that children have a greater preference for sweet and salty foods than adults [14], making them particularly vulnerable in an environment in which salt and sugar dominate the food supply.

Food patterns are established in childhood, and the “family environment” and parents’ behavior (as food choices or feeding practices) determine the children's food preferences and diet [15]. Given the knowledge of the current excessive sodium intake in Spanish children and the benefits of decreasing salt intake from an early age to reduce the likelihood of a diet high in salt during adulthood or to prevent cardiovascular disease [16], it is useful to try to understand current patterns involving schoolchildren and their environment regarding behaviors related to salt intake. These behaviors include the use of discretionary salt (adding salt at the table or when cooking) and checking the sodium content on food labeling [17]. Furthermore, given children’s vulnerability to developing a preference for salty foods, it would be interesting to analyze whether these behaviors are related to children’s greater preference for salty foods.

Currently, there are no available data about the behaviors of Spanish parents and their children related to the use of discretionary salt and the relationship between parental behaviors to their children’s sodium intake. Our main objective is to determine parents’ and their own children’s salt-related behaviors and their association with the children’s 24 h urinary sodium excretion (UNa-24h). Our secondary objectives are to examine the association between parental salt-related behaviors and (1) high sodium excretion among children, (2) children’s table salt use, and (3) children’s preference for “salty foods” in the context of Spanish schoolchildren.

## Material and methods

### Study design

A convenience sample was used from an ongoing cross-sectional trial registered at clinicaltrials.gov as NCT03465657. The main aim of this study is to establish the average sodium intake in Spanish schoolchildren according to sodium excretion in a 24 h urine sample and analyze the relationship between salt intake and health parameters. Another goal is to identify the use of discretionary salt in parents and children. The data reported in this manuscript were collected between February 2014 and February 2018. The study design has been previously described in other papers [10,18]. The study protocol was approved by the Ethics Committee of the San Carlos Hospital (Ref 12/319-E and 15/522-E), which is part of the Complutense University of Madrid [19]. Coding was used to identify samples and forms.

### Data collection and subjects

**Fig 1:**
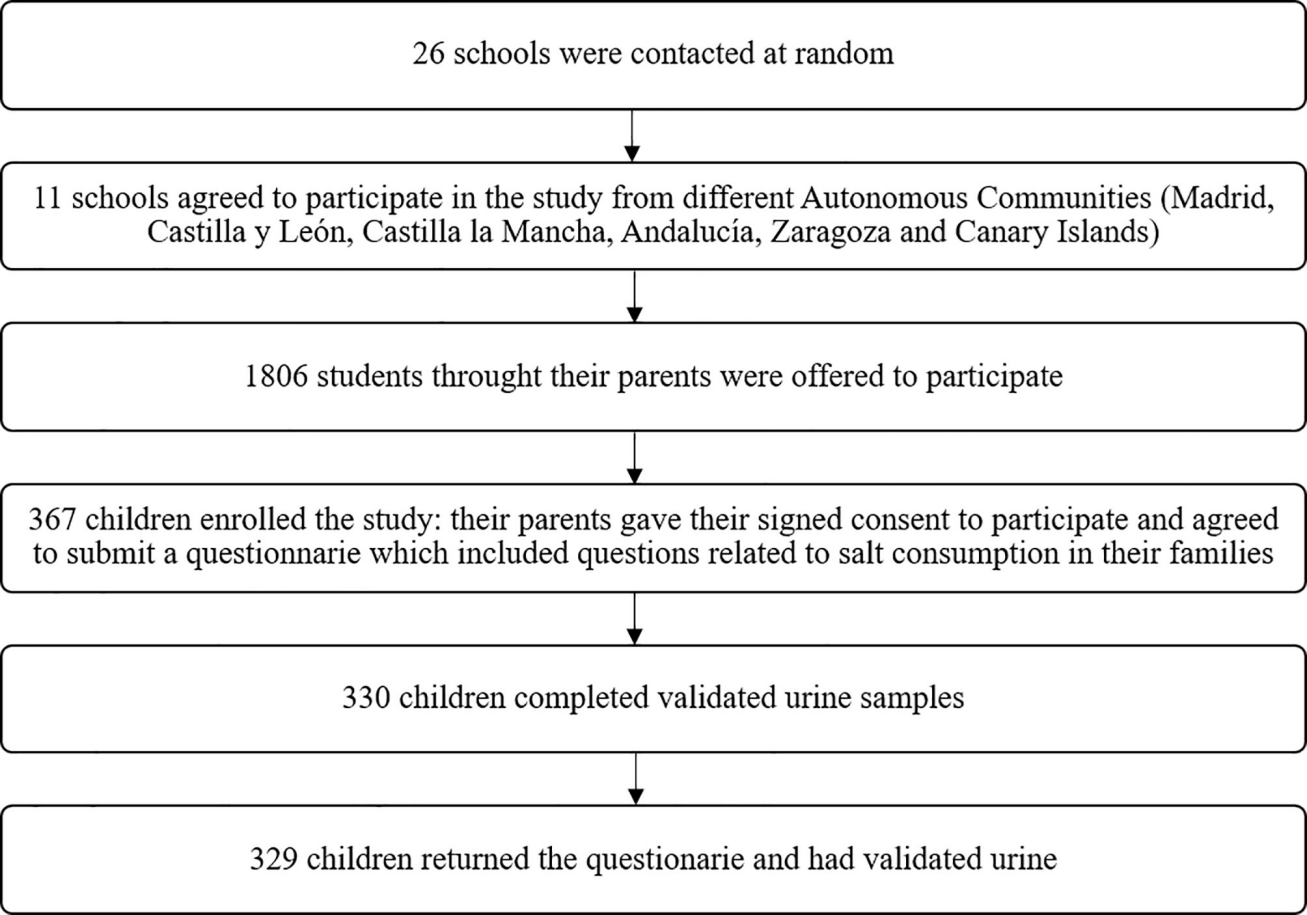
Recruitment of the population.

This convenience sample involved primary schools from six Spanish provinces (Madrid, Córdoba, Segovia, Ciudad Real, Zaragoza, and Tenerife), including the capital of the province and a semi-urban/rural city (<50000 inhabitants). A number was assigned to the schools, and schools were chosen randomly from each location. The random selection of schools was conducted using Microsoft Office Excel 2013 and the RANDBETWEEN function [20]. Of the twenty-six schools contacted by telephone, eleven accepted the invitation to be included in the study (Fig 1).

First, the schools were contacted by telephone. Once the Director agreed to the school's participation, an appointment was arranged with the parents, who were invited to an informative talk during which the details of the study were explained. Once any questions posed by the parents were answered, the interested parents signed a written informed consent form. On the second day, the researchers visited the school to collect the questionnaires completed by the parents and children. Subsequently, the children gave a urine sample, and several anthropometric measurements were made. Of the 1806 children and their own parents that were offered to participate, 367 parents gave their informed consent and enrolled with their children in the study (20.3%).

The inclusion criteria were schoolchildren aged 7–11 years, attending between the second and fifth grades of primary school, and compliance with the informed consent via parents or legal guardians. The exclusion criteria were absence on the day of the visit to the school, having a disease that could affect the excretion of sodium (metabolic or chronic diseases, such as diabetes, kidney disease, or liver disease), altered eating habits (when the child has to modify his or her diet in a timely manner for some reason, e.g., a soft diet), and not providing a urine sample.

### Sociodemographic and anthropometric data

Parents completed a self-administered questionnaire at home about the children and family health status. The questionnaire also included sociodemographic data, such as the working situation of the father and mother (details of the questionnaire described below).

Anthropometric measurements were carried out in the schools. Weight and height were determined with a digital balance (range 0.1–150 kg, accuracy 100 g; Alpha; Seca, Igni, France) and a digital stadiometer (70–205 cm, 1 mm; Harpenden Pfifter, Carlstadt, NJ, USA), respectively. For these measurements, the children were barefooted and wore only their underwear. For the analyses, the mean value of three measurements was used. Their body mass index (BMI) was then calculated.

### Twenty-four-hour urine sample

A 24-hour urine sample was analyzed to study the urinary parameters. To ensure compliance with 24-hour urine collection, the schoolchildren and their parents were instructed in the collection procedure and given written instructions. The children were requested to urinate at 8 p.m. (start of the collection), and this urine sample was excluded. Then, the subsequent voiding was included in the collection. From that moment onward, all urine was collected to obtain the complete sample at the scheduled time (24 hours after the start time of the collection), with instructions that the last urine sample be collected at the same hour as the first sample. This protocol was adapted from Neubert et al. [21]. The 24-hour sampling began Saturday night and continued until Sunday at the same time to facilitate the collection of urine samples on a non-teaching day. Parents were asked to write down any urinations that were not collected during the 24 hours.

All urine was stored in a 2 L plastic container without preservatives. After receiving the urine, the volume of the 24-hour urine sample was calculated, and the urine was stored in 100 mL containers at a temperature below 12°C before being transferred to the laboratory.

Urinary sodium excretion was quantified using an indirect potentiometer, which had selective solid membranes for each ion and was connected to an AU 5400 Autoanalyzer (Olympus, Mishima, Japan): the coefficient of variation (CV) was 1.0 for sodium [22]. Creatinine levels were determined according to a modification of the Jaffe reaction using the same apparatus. The color intensity was measured at 520 nm (CV = 2.8) [23]. Creatinine was expressed in mg/day and converted to mmol/kg/day with the following factor: 1 mg of creatinine × 0.0088 = 1 mmol of creatinine. We used a cut-off limit taken from Remer et al. for creatinine [24] to identify under-collected 24 h urine samples. Collections were considered incomplete if (1) there were self-reported missing voids (n = 19), (2) urinary creatinine < 0.1 mmol/kg/day (n = 18) [24], and (3) volume < 300 mL (n = 0) [25].

### Behaviors related to salt consumption

Parents completed a self-administered questionnaire at home about behaviors concerning salt consumption. They answered eight closed questions regarding (1) the use of salt by the person in charge of cooking; (2) the use of table salt after cooking by the father (3) and mother; (4) the frequency of checking the salt content on food labels; (5) the type of salt used; (6) the availability of a salt shaker on the table; (7) the free use of the salt shaker by the children; (8) and the schoolchildren's preference for salty foods. S1 File shows the questionnaire used with all answer options.

### Statistical processing of data

The results were summarized as the mean ± standard deviation (SD), medians with the interquartile range (IQR), or proportions (for categorical variables). The Kolmogorov–Smirnov test was used to assess the assumption of normality. To compare the main baseline characteristics of the participants according to sex, we used the Student’s t-test on homogeneous variables and the Mann–Whitney U test on non-parametric variables. The χ2 test was used for categorical variables, and the Spearman correlation (*r_S_*) was employed to assess the relationship between UNa-24h and BMI and age by sex. UNa-24h was expressed in mg/day and salt equivalent in g/day, and it was studied according to its relationship with different behaviors. Subjects who did not answer a question were excluded from the analysis of that question but not from the overall study. Because not all data followed a normal distribution, the median test for k independent samples was used to compare sodium excretion between different groups regarding dietary behaviors related to salt intake to identify differences in sodium excretion [26], and the Levene homoscedasticity test was used to determine whether variance differed significantly between groups. Pairwise multiple comparisons were used to observe the differences between groups.

Afterward, logistic regression models with calculations of the corresponding odds ratio (OR) and 95% confidence intervals (CI) were used to examine the possible association between parents’ and children’s behaviors related to salt (independent variables) with children’s sodium excretion above the median or with the use of table salt by children (dependent variables). The responses in the questions about behaviors related to salt were consolidated into two groups: ‘only if it is tasteless/sometimes’ and ‘always’ were combined into one category to compare with ‘never’. The preference for salty foods was consolidated into ‘somewhat salty/salty food’ versus ‘not salty food’, and the child’s use of table salt was grouped into ‘sometimes/always’ (when the response was from ‘Once a day’ to ‘1–3 times in a month’) versus ‘never’ (when the response was ‘Never, less than once in a month’). We evaluated the associations using three models: (a) a basic model that was not adjusted, (b) a second model that accounted for the sex, age, and BMI of the children, and (c) a third model that included the second model’s considerations plus the simultaneous effect of all explanatory variables to analyze those that had the most significant influence. Subsequently, to study the children’s preference for salty or medium-salt-content foods, we used multinomial logistic regression models adjusted by the variables described above in the three models. A p-value of p < 0.05 was considered statistically significant. The evaluation of the data obtained was completed with the SPSS® version 24.0 statistical software.

## Results

Of the 367 children who enrolled in the study, three did not return the behavior questionnaire completed by their family. For the urine study, 19 participants did not complete the 24-hour collection of urine (the samples were lost during collection), while a further 18 participants had creatinine excretion rates below the cut-limit of 0.1 mmol/kg/day, resulting in a final sample of 329 children with valid urine samples and completed salt behavior questionnaires (Fig 1).

The mean age of children was 9.0 ± 1.2 years old, with 52% boys, and the mean UNa-24h was 3133 ± 1194 mg/24 h (7.8 ± 3.0 g/day of salt). There were no significant differences between the sexes in the sociodemographic characteristics shown in Table 1, but there were differences in UNa-24h (p < 0.01) and creatinine/weight (p < 0.001), which were both higher in boys than in girls. Furthermore, there were a positive correlations between sodium excretion and BMI and age in boys (*r_S_* = 0.336, p < 0.001; *r_S_* = 0.174, p < 0.05, respectively) and girls (*r_S_* = 0.193, p < 0.01; *r_S_* = 0.336, p < 0.05, respectively).

**Table 1:** Participants’ demographic, anthropometric, and urinary parameters by children’s sex (mean ± SD or %).

Variables	Subcategories	Total children (n = 329)	Girls (n = 157)	Boys (n = 172)	*p*^a^ value
**Children**					
Age (years)		9.0 ± 1.2	9.0 ± 1.2	8.9 ± 1.2	0.664
**Geographical area (%)**					0.656
	<50000 inhabitants	46.5	45.2	47.7	
	>50000 inhabitants	53.5	54.8	52.3	
**Anthropometric data**					
Weight (kg)†		35.6 ± 8.5	35.8 ± 8.8	35.4 ± 8.1	0.654
Height (cm)		137.3 ± 8.9	137.0 ± 9.5	137.6 ± 8.2	0.939
BMI (kg/m^2^)†		18.7 ± 3.2	18.9 ± 3.2	18.6 ± 3.2	0.234
**Urinary parameters**					
Volume 24 h (mL/24 h)†		907.2 ± 297.2	886.5 ± 284.9	926.1 ± 307.7	0.228
Creatinine/weight (mg/kg)†		20.6 ± 4.3	19.4 ± 4.0	21.6 ± 4.4	**0.000**
UNa-24h (mEq/24 h)		135.9 ± 51.9	127 ± 44.4	144.1 ± 56.8	**0.007**
UNa-24h (mg/24 h)		3133 ± 1194	2921 ± 1022	3314 ± 1306	**0.007**
Salt (g/day)		7.9 ± 3.0	7.4 ± 2.6	8.4 ± 3.3	**0.007**
Na/Creatinine (mg/mg)		4.4 ± 1.4	4.4 ± 1.4	4.4 ± 1.5	0.785
**Parents**					
Age (years)†		42.0 ± 5.2	42.7 ± 5.1	41.5 ± 5.3	**0.008**
**Father's employment status (%)**					0.659
	Non-paid work/no work	11.9	12.7	11.0	
	Private company	65.3	62.4	68.0	
	Official position	16.1	17.8	14.5	
	Retired	1.2	0.6	1.7	
	DK, NA	5.5	6.4	4.7	
**Mother's employment status (%)**					0.195
	Non-paid work/no work	36.8	32.5	40.7	
	Private company	45.0	48.4	41.9	
	Official position	16.4	18.5	14.5	
	Retired	0.9	0.0	1.7	
	DK, NA	0.9	0.6	1.2	

† Data do not follow a normal distribution. BMI: Body Mass Index. DK, NA: do not know, no answer. UNa-24h: 24 h urinary sodium excretion.

^a^ Significant difference according to sex group, as shown by the Student’s t-test for continuous and parametric variables, the Mann–Whitney U test for nonparametric variables (†), and the chi-square test among the groups. Significant differences are bolded.

### Use of discretionary salt and other behaviors by the families

Ninety-two percent of the families reported adding salt to their meals while cooking. Forty-three percent of the fathers replied that they never added salt at the table compared with 48% of the mothers (Table 2). More than half of the families reported using iodized salt (53%). A total of 59% of parents never checked the sodium content on food labels. Most of the families (94%) reported never having a salt shaker on the table, and 80% of the children reported never adding salt to food after it is cooked.

**Table 2:** Frequency of dietary behaviors related to salt intake and their relationship with sodium excretion (mg/day) in 24-hour urine in Spanish schoolchildren.

Behaviors				UNa-24h (mg/24h)		Salt equivalent (g/day)		
Questions	Subcategories	n	%	Mean ± SD	Median (IQR)	Mean ± SD	Median (IQR)	p value†
**In your home, do you add salt to food while cooking?**	No	27	8.4	3218 ± 1381	3082 (2001–4209)	8.2 ± 3.5	7.8 (5.1–10.7)	0.910
	Yes	295	91.6	3126 ± 1179	3036 (2323–3772)	7.9 ± 3.0	7.7 (5.9–9.6)	
**Do you add salt to food when you eat it after it is cooked? (Father)**	Never	129	43.0	3008 ± 1332	2806 (2116–3726)	7.6 ± 3.4	7.1 (5.4–9.5)	0.232
	Only if it is tasteless	157	52.3	3166 ± 1021	3151 (2461–3772)	8.0 ± 2.6	8.0 (6.3–9.6)	
	Always	14	4.7	3343 ± 1228	3289 (2208–4025)	8.5 ± 3.1	8.4 (5.6–10.2)	
**Do you add salt to food when you eat it after it is cooked? (Mother)**	Never	157	48.3	3064 ± 1313	2875 (2208–3772)a	7.8 ± 3.3	7.3 (5.6–9.6)a	**0.048**
	Only if it is tasteless	153	47.1	3152 ± 1096	3128 (2392–3772)	8.0 ± 2.8	7.9 (6.1–9.6)	
	Always	15	4.6	3384 ± 941	3174 (2783–3910)b	8.6 ± 2.4	8.1 (7.1–9.9)b	
**In your home, do you routinely check food labels for salt content?**	Never	192	59.1	3069 ± 1124	3071 (2289–3738)	7.8 ± 2.9	7.8 (5.8–9.5)	0.830
	Sometimes	104	32.0	3141 ± 1224	2990 (2254–3876)	8.0 ± 3.1	7.6 (5.7–9.8)	
	Always	29	8.9	3199 ± 1262	3174 (2208–4209)	8.1 ± 3.2	8.1 (5.6–10.7)	
**In your home, is the salt shaker on your table for anyone who wants it?**	Never	308	94.2	3094 ± 1191	2990 (2243–3749)a	7.9 ± 3.0	7.6 (5.7–9.5)a	**0.025**
	Sometimes	13	4.0	3761 ± 1084	3887 (3197–4140)b	9.6 ± 2.8	9.9 (8.1–10.5)b	
	Always	6	1.8	3320 ± 1528	3370 (1817–4209)	8.4 ± 3.9	8.6 (4.6–10.7)	
**Does your child prefer not salty or very salty food?**	Not salty	22	6.7	3078 ± 915	2956 (2691–3381)	7.8 ± 2.3	7.5 (6.8–8.6)	0.529
	Somewhat salty	269	82.3	3125 ± 1245	3036 (2231–3818)	7.9 ± 3.2	7.7 (5.7–9.7)	
	Very salty	36	11.0	3134 ± 970	3151 (2461–3864)	8.0 ± 2.5	8.0 (6.3–9.8)	
**How often does your child add salt to food after it is cooked?**	Never, less than once in a month	264	80.2	3381 ± 1850	3105 (1875–4888)	8.6 ± 4.7	7.9 (4.8–12.4)	0.901
	1–3 times in a month	28	8.5	3332 ± 1780	3289 (1426–5244)	8.5 ± 4.5	8.4 (3.6–13.3)	
	Once a week	8	2.4	2921 ± 763	2829 (2208–3726)	7.4 ± 1.9	7.2 (5.6–9.5)	
	2–3 times in a week	7	2.1	2865 ± 1011	3013 (2047–3887)	7.3 ± 2.6	7.7 (5.2–9.9)	
	4–6 times in a week	3	0.9	3134 ± 665	3163 (2565–3715)	8.0 ± 1.7	8.0 (6.5–9.4)	
	Once a day	7	2.1	3370 ± 1008	3232 (2783–4014)	8.6 ± 2.6	8.2 (7.1–10.2)	
	> Once a day	4	1.2	3090 ± 1190	2979 (2323–3761)	7.9 ± 3.0	7.6 (5.9–9.6)	
	DK, NA	8	2.4	3459 ± 1847	3381 (1886–4658)	8.8 ± 4.7	8.6 (4.8–11.8)	
**In your home, do you use iodized salt or regular salt?**	Regular salt	120	42.4	3009 ± 1202	2829 (2208–3634)a	7.6 ± 3.1	7.2 (5.6–9.2)a	**0.007**
	Iodized salt and regular salt	4	1.4	2266 ± 1288	2001 (1403–3128)	5.8 ± 3.3	5.1 (3.6–7.9)	
	Iodized salt	151	53.4	3277 ± 1156	3347 (2438–4002)b	8.3 ± 2.9	8.5 (6.2–10.2)b	
	Others (ecological salt)	8	2.8	2923 ± 1192	2933 (2507–3588)	7.4 ± 3.0	7.4 (6.4–9.1)	

DK, NA: do not know, no answer. IQR: interquartile range. UNa: urinary sodium excretion.

†Sodium excretion for each question was compared using the median test to compare medians between groups. Values in the same category suffixed with a or b are significantly different from each other at p < 0.05 when compared by pairwise multiple comparisons. Significant differences are bolded.

When comparing the use of table salt between fathers and mothers, it was observed that both parents tended to have the same answers (S1 Fig): when the mother reported adding salt ‘always when she eats after the food is cooked’, a higher proportion of fathers did so as well (82%). The same pattern occurred when the mother reported only adding salt if the food was tasteless (74% of the fathers answered the same) or never added it (62% of the fathers did the same).

### Dietary behaviors and their relationship with UNa-24h in Spanish schoolchildren

**Table 2** also describes the differences in 24-hour urine sodium excretion related to behavioral questions about salt. The schoolchildren did not show differences in the urinary sodium for five of the eight questions related to the parents or children. Differences in sodium excretion were observed according to the use of table salt by the mother (p < 0.05), the presence of the salt shaker on the table (p < 0.05), and the type of salt used (p < 0.01). The children of mothers who reported never adding salt to food after it is cooked had lower sodium excretion than those who reported always adding salt. Further, in families in which the salt shaker was sometimes on the table, schoolchildren excreted more sodium than those who never had it present. In addition, in families using iodized salt, schoolchildren excreted more sodium than children in families using regular salt.

In **Table 3**, the multivariate analysis results show that the use of salt at the table by the father or mother, the presence of the salt shaker on the table, the use of iodized salt and the use of table salt by the children were positively associated with an increased risk of excreting sodium over the median after adjusting for sex, age, and BMI. Further, the use of iodized salt remained significant after taking into account the simultaneous effect of all the variables.

**Table 3:** Logistic regression models. Odds ratios and 95% confidence intervals for the presence of sodium excretion greater than 3048 mg/day (50th percentile).

Predictor Variables		Model 1		Model 2		Model 3	
Questions	Groups	OR (CI 95%)	p value	OR (CI 95%)	p value	OR (CI 95%	p value
In your home, do you add salt to the food while cooking? ^a^	Sometimes/Always	0.974 (0.443–2.143)	0.947	1.246 (0.545–2.847)	0.602	1.039 (0.380–2.846)	0.940
Do you add salt to food when you eat it after it is cooked? (Father) ^a^	Only if it is tasteless /Always	1.629 (1.028–2.583)	**0.038**	1.719 (1.061–2.785)	**0.028**	1.385 (0.780–2.460)	0.267
Do you add salt to food when you eat it after it is cooked? (Mother) ^a^	Only if it is tasteless /Always	1.617 (1.043–2.507)	**0.032**	1.663 (1.052–2.629)	**0.029**	1.277 (0.727–2.241)	0.395
In your home, do you routinely check food labels for salt content? ^a^	Sometimes/Always	0.925 (0.595–1.44)	0.731	0.876 (0.551–1.392)	0.575	0.969 (0.567–1.657)	0.909
In your home, is the salt shaker on your table for anyone who wants it? ^a^	Sometimes/Always	3.849 (1.249–11.859)	**0.019**	4.730 (1.473–15.188)	**0.009**	.3844 (0.978–15.101)	0.054
Does your child prefer not salty or very salty food? ^b^	Somewhat salty/Very salty	1.273 (0.534–3.035)	0.586	1.445 (0.587–3.557)	0.423	1.651 (0.523–5.212)	0.393
How often does your child add salt to the food after it is cooked? ^a^	Sometimes/Always	1.794 (0.995–3.236)	0.052	1.963 (1.060–3.635)	**0.032**	1.485 (0.728–3.027)	0.277
In your home, do you use iodized salt or regular salt? ^c^	Iodized salt and regular salt	0.425 (0.043–4.184)	0.463	0.638 (0.063–6.445)	0.703	-^d^	-^d^
	Iodized salt	1.901 (1.203–3.005)	**0.006**	1.919 (1.187–3.103)	**0.008**	1.710 (1.007–2.904)	**0.047**
	Others (ecological salt)	0.849 (0.23–3.142)	0.807	0.873 (0.23–3.316)	0.841	1.235 (0.274–5.571)	0.783

Dependent variable: UNa-24h ≥ 50th percentile.

^a^The reference is ‘never’.

^b^The reference is ‘not salty food’.

^c^The reference is ‘regular salt’.

^d^Data was removed because it was statistically unreliable.

Model 1: not adjusted. Model 2: adjusted for age, BMI, and sex. Model 3: model 2 plus the rest of the predictor variables (In your home, do you add salt to the food while cooking? Do you add salt to food when you eat it after it is cooked? (father, mother); In your home, do you routinely check food labels for salt content? Does your child prefer not salty or very salty food? How often does your child add salt to the food after it is cooked? In your home, do you use iodized salt or regular salt?). Significant differences are bolded.

### Behaviors and preferences of children and their relationship with the behaviors of parents

The association between the salt use behaviors of the family and the use of table salt by the children is shown in **Table 4**. The use of table salt by father or mother was positively associated with a higher risk of the use of table salt ‘sometimes or always’ by the children after adjusting for sex, age and BMI (p < 0.01). As opposed to never having the salt shaker on the table, its presence on some occasions or all the time was also positively associated with an increased risk of the use of table salt by the children after adjusting for sex, age, and BMI (p < 0.001). After adjusting for all factors simultaneously, only the use of table salt by the father remained significant (p < 0.05).

**Table 4:** Logistic regression models. Odds ratios and 95% confidence intervals for the child's use of table salt sometimes/always versus never.

Predictor Variables		Model 1		Model 2		Model 3	
Questions	Groups	OR (CI 95%)	p value	OR (CI 95%)	p value	OR (CI 95%)	p value
In your home, do you add salt to the food while cooking? ^a^	Sometimes/Always	1.595 (0.459–5.541)	0.462	0.667 (0.190–2.340)	0.527	1.329 (0.255–6.910)	0.736
Do you add salt to food when you eat it after it is cooked? (Father) ^a^	Only if it is tasteless/Always	5.396 (2.441–11.930)	**0.000**	5.231 (2.359–11.603)	**0.000**	3.813 (1.553–9.361)	**0.003**
Do you add salt to food when you eat it after it is cooked? (Mother) ^a^	Only if it is tasteless/Always	1.635 (3.094–5.853)	**0.001**	3.231 (1.697–6.151)	**0.000**	2.076 (0.977–4.410)	0.057
In your home, do you routinely check food labels for salt content? ^a^	Sometimes/Always	1.206 (0.674–2.158)	0.528	1.159 (0.643–2.089)	0.623	1.147 (0.576–2.283)	0.697
In your home, is the salt shaker on your table for anyone who wants it? ^a^	Sometimes/Always	5.292 (1.998–14.016)	**0.001**	5.604 (2.081–15.092)	**0.001**	2.133 (0.662–6.877)	0.205
Does your child prefer not salty or very salty food?^b^	Somewhat salty/Very salty	2.133 (0.483–9.426)	0.318	2.037 (0.458–9.052)	0.350	2.027 (0.237–17.353)	0.519
In your home, do you use iodized salt or regular salt?^c^	Iodized salt and regular salt	2.105 (0.208–21.301)	0.528	1.846 (0.178–19.130)	0.607	9.192 (0.664–127.299)	0.098
	Iodized salt	1.641 (0.885–3.042)	0.116	1.763 (0.940–3.307)	0.077	1.409 (0.703–2.823)	0.334
	Others (ecological salt)	0.702 (0.084–5.858)	0.774	0.648 (0.077–4.472)	0.690	0.634 (0.068–5.866)	0.688

Dependent variable: the use of table salt sometimes/always.

^a^The reference is ‘never’.

^b^The reference is ‘not salty food’.

^c^The reference is ‘regular salt’.

^d^Data was removed because it was statistically unreliable.

Model 1: not adjusted. Model 2: adjusted for age, BMI, and sex. Model 3: Model 2 plus the rest of the predictor variables (In your home, do you add salt to the food while cooking? Do you add salt to food when you eat it after it is cooked? (Father, mother); In your home, do you routinely check food labels for salt content? Does your child prefer not salty or very salty food? In your home, do you use iodized salt or regular salt?). Significant differences are bolded.

**Table 5** shows which behaviors are related to children's preference for somewhat salty or very salty foods over non-salty food. The father's use of table salt was associated with a higher risk of preferring somewhat salty or very salty foods by the schoolchildren (p < 0.05), the use of table salt by the children was associated with a higher risk of preferring salty food (p < 0.05), while checking the sodium content on food labels was associated with a lower risk of having a preference for salty foods (p < 0.05) after adjusting for age, sex, and BMI. After adjusting for all factors simultaneously, no one remained significant.

**Table 5:** Multinomial logistic regression models. Odds ratios and 95% confidence intervals for children's preference for somewhat salty/very salty foods.

		Model 1				Model 2				Model 3			
Predictor Variables		Medium salt content		Salty food		Medium salt content		Salty food		Medium salt content		Salty food	
Questions	Groups	OR (CI 95%)	p value	OR (CI 95%)	p value	OR (CI 95%)	p value	OR (CI 95%)	p value	OR (CI 95%)	p value	OR (CI 95%)	p value
In your home, do you add salt to the food while cooking?^a^	Sometimes/Always	3.034 (0.928–9.923)	0.066	2.588 (0.519–12.913)	0.246	2.772 (0.833–9.219)	0.096	2.124 (0.408–11.053)	0.371	3.818 (0.981–14.863)	0.053	3.207 (0.390–26.359)	0.278
Do you add salt to food when you eat it after it is cooked? (Father)^a^	Only if it is tasteless/ Always	2.777 (1.083–7.122)	**0.034**	3.818 (1.192–12.231)	**0.024**	2.697 (1.044–6.969)	**0.041**	3.330 (1.021–10.862)	**0.046**	2.341 (0.669–8.200)	0.183	1.639 (0.343–7.826)	0.536
Do you add salt to food when you eat it after it is cooked? (Mother)^a^	Only if it is tasteless/ Always	2.205 (0.862–5.636)	0.099	2.8 (0.910–8.611)	0.072	2.234 (0.871–5.729)	0.094	3.054 (0.978–9.534)	0.055	1.129 (0.330–3.859)	0.847	1.141 (0.248–5.247)	0.865
In your home, do you routinely check food labels for salt content?^a^	Sometimes/Always	0.473 (0.195–1.146)	0.097	0.305 (0.101–0.921)	**0.035**	0.453 (0.185–1.109)	0.083	0.280 (0.090–0.866)	**0.027**	0.665 (0.222–1.994)	0.466	0.293 (0.072–1.199)	0.088
In your home, is the salt shaker on your table for anyone who wants it?^a^	Sometimes/Always	1.157 (0.145–9.237)	0.89	2.625 (0.274–25.140)	0.402	1.164 (0.145–9.354)	0.886	2.710 (0.274–26.795)	0.394	- ^c^	- ^c^	- ^c^	- ^c^
How often your children add salt to the food after it is cooked?^a^	Sometimes/Always	1.667 (0.374–7.434)	0.503	8.382 (1.669–42.103)	0.**010**	1.615 (0.361–7.232)	0.531	7.753 (1.523–39.473)	0.**014**	1.306 (0.153–11.152)	0.807	8.029 (0.824–78.283)	0.073
In your home, do you use iodized salt or regular salt?^b^	Iodized salt	2.012 (0.767–5.281)	0.155	2.449 (0.771–7.778)	0.129	2.123 (0.798–5.646)	0.131	3.112 (0.949–10.197)	0.061	2.577 (0.802–8.277)	0.112	3.169 (0.760–13.218)	0.113

Dependent variable: children's preference for somewhat salty/very salty foods.

^a^The reference is ‘never’.

^b^The reference is ‘regular salt’.

^c^Data was removed because it was statistically unreliable.

Model 1: not adjusted. Model 2: adjusted for age, BMI, and sex. Model 3: Model 2 plus the rest of the predictor variables (In your home, do you add salt to the food while cooking? Do you add salt to food when you eat it after it is cooked? (Father, mother); In your home, do you routinely check food labels for salt content? Does your child prefer not salty or very salty food? How often does your child add salt to the food after it is cooked? In your home, do you use iodized salt or regular salt?). Significant differences are bolded.

## Discussion

This study provides a novel understanding of the salt-specific behaviors of Spanish families and their association with the current levels of sodium excretion in the children, studying the parental reported behaviors compared to sodium excretion of their own children. On average, schoolchildren consumed 7.9 g/day of salt (only 15.2% of children had a salt intake below 5 g/day). This amount is similar for Portuguese schoolchildren [27], but in children from Italy [28], Germany [29], or the United Kingdom [30], the intake is lower. These differences may be due to different age ranges in the studies or differences in the quality control to exclude urine collections (for example, Cotter et al. [27] used a volume < 400 mL urine in 24 h). Besides, it could be affecting the day of the collection since, in other populations, salt intake was higher on the weekend vs. weekdays [31,32]. There was a relationship between the urinary sodium excretion in children and the use of table salt by parents or children, the use of iodized salt by the families, and the presence of the salt shaker on the table. With regard to the use of table salt, schoolchildren with families who sometimes or always had the salt shaker on the table or with fathers or mothers adding salt at the table were more likely to use table salt. Children preferred salty foods when they added salt to their food or when their father sometimes or always added table salt, and they preferred non-salty foods when the parents reported checking labels. We looked into behaviors that can be modified by educational interventions aimed at parents and children in order to change the levels of excessive salt intake in the family and childhood environment.

The proportion of children who use table salt in our study (17%) and parents who reported placing a salt shaker on the table at mealtimes (6%) was lower than in Australian children and parents (32% and 45% respectively) [33]. Meanwhile, slightly more than 50% of Spanish fathers and mothers use table salt (52–57%), a similar proportion to the 54% of the Australian parents. Differently, 92% of Spanish parents reported adding salt during cooking, a result higher than 67% of Australian parents, 65%-71% of Belgian adults or 77%-85% of Norwich adults, and it was more similar to that found it in Slovaks adults (91% 98%) [33,34]. The differences between Spanish parents and the adults of other European countries could be due to different samples (mean age in Spanish parents: 42 ± 5 years; mean age in the adults studied in the De Keyzer et al. study: 53–55 years) [34].

Regarding the type of salt used, 53% of the families consumed iodized salt, followed by those who consumed regular salt (42%). Our result is in line with the results found it in other studies, in which it was observed that 44% and 69% of the Spanish adult and child population, respectively, consumed iodized salt [35,36]. In addition, in our study, families who reported using iodized salt had children with higher sodium intake and risk of sodium excretion above the median than families who used regular salt. In contrast to these results, in the study of Nazeri et al. [37], the behavior of mothers regarding the use of iodized salt was not associated with the ingestion of salt by their relatives. WHO stated that ‘Ministries of health should make sure that the message to consume iodized salt does not promote excessive salt consumption’[38]. One possible cause of this relationship is that iodized salt is potentially considered less dangerous or healthier than regular salt because its iodine content has been promoted as a benefit [39], perhaps leading people to use it more indiscriminately. It is known that iodized salt is a vehicle for introducing iodine, which is in line with iodine intake control policies that aim to ensure adequate intake of this essential mineral—this could be useful in this population [40–42]. As Vila et al. said, there are no national initiatives in Spain regarding the consumption of iodized salt at the moment, so it would be advisable to implement them [42]. In the present study, the use of iodized salt was associated with higher sodium intake. It would be advisable to establish policies about iodine while monitoring the population’s sodium intake [39,42] to provide guidelines to limit the use of discretionary salt, promote the use of iodized salt, and encourage people to choose foods that have less hidden salt.

Regarding other behaviors, the use of salt while cooking at home was not related to the sodium excretion of the children. These results differ from those found in a study on Australian children [43], who found an inverse relationship between the addition of cooking salt by parents and the sodium excretion of their children. As mentioned by Service et al. [43], other food choices (i.e., processed food vs. unprocessed food, the frequency of parents cooking at home vs. eating out, or the seasoning of meals) could explain the lack of positive association. However, the odds for children having a sodium excretion above the median (3,048 mg/d) were higher for parents who reported adding table salt to food ‘always or only if it is tasteless’, for children who used table salt or when the salt shaker was accessible on the table. Sodium intake by schoolchildren has previously been associated with parental sodium intake, probably because of closeness in dietary habits or behaviors [27,43].

In this study, roughly 40% of the parents reported checking the sodium content on food labels (Table 2). However, this behavior was not associated with the excretion of less sodium of their children. In adults, it has been observed that consulting the salt content on the label is related to lower sodium intake [44,45]. However, no such association has been found in other studies [46,47]. In this study the lack of association could be because parents were not purchasing low-salt foods, or parents may only look at the salt content of foods that taste salty but not at other foods that contain salt but do not taste salty (such as breakfast cereals); alternatively, parents may not understand the label and maybe they don’t know how to read sodium information on food labels, or just there are other factors not picked up that are affecting such as a high frequency of eating out. Nevertheless, encouraging people to look at the label or making it easier for them to do so is essential [48] and it is important to establish early dietary practices related to salt intake. A recent report from the National Academies of Sciences, Engineering, and Medicine recommends reducing intakes of sodium in children if above 1,500 mg/d (children aged 4–8 years) or 1,800 mg/d (children aged 9–13 years) to reduce chronic disease risk [49].

Also, children’s salt taste preference was not associated with children’s 24 h urinary sodium excretion. Other studies have found that children who liked salty foods consumed more salt. For example, in a study by Matsuzuki et al. [50], children who preferred salty foods, as observed by their mothers, consumed more salt. Similarly, in a study by Mennella et al. [14], it was observed that the children’s preference for salty foods and the reported sodium intake were related. However, in our study, we found no association between the preference for salty foods and sodium excretion. One possible explanation is that these children may not eat salty foods frequently, even though they prefer them. Additionally, it should be borne in mind that part of the dietary sodium found in foods is not considered to have a salty taste, for example, saltiness in breakfast cereals is not usually obvious to the taste [51,52] and parents could be unaware of the amount of sodium in these foods. Moreover, the saltiness of food affects the preference we have for food, depending on the type of food [53], and children may like some salty foods but not all of them.

About the relationship of behaviors between parents and their child, the use of table salt by father or mother was associated with table salt use by children. Both the father and mother can potentially influence salt intake in schoolchildren because of their involvement in early feeding [15]. Moreover, the behaviors of one parent were related to those of the other parent (S1 Fig), and this could be associated with the adaptation of family members to their family environment. In addition, children who sometimes or always had a salt shaker nearby on the table were more likely to use table salt sometimes or always. The use of table salt by the parents and the presence of a salt shaker may be associated with an adaptation by children to prefer saltier foods and to use table salt.

Regarding the preference for salty foods, we found a relationship between the use of table salt by the father and the use of table salt by the children with children's preference for salty foods. Different factors could explain this relationship, maybe children living in a family environment in which table salt is used by the father could have more exposure to table salt and salty foods and therefore prefer them to non-salty foods since liking a salty taste is determined by exposure to salty foods [51,54]. Also, this preference for salty foods could be due to the presence of genetic components of taste or with the imitation of parents’ food choices [55]. On the other hand, parental label viewing of sodium content ties into children’s preference for low-salt foods. One explanation could be that parents who read the sodium value on the label may have more nutritional knowledge, because nutritional knowledge has been positively related to labeling reading and inversely related to the use of discretionary salt in adults [56–58].

It is known that there is an excessive intake of sodium in Spanish adults and children [7,10]. The primary sources of dietary sodium (excluding salt added at the table or during cooking) in the Spanish population are meat, meat products, cereals, and cereal products [6,7,10]. Further, most dietary sodium comes from discretionary foods (foods and beverages that are high in saturated fats, sugars, salt, and/or alcohol and nutrient-poor) [18]. Besides that, in Spanish adults, discretionary salt intake contributes to the total sodium intake with 21.0 ± 10.3% (1.5 ± 0.8 g/day of salt) [59]. The most likely explanation for the high salt intake in Spain could be barriers to behavioral change. The adverse nature of the food environment, which includes food and beverage advertising [60], high salt content in processed foods [18], and labeling that is challenging to understand [48], could impede the reduction of salt intake. Among the strategies to reduce sodium consumption in the population, agreements have been established with the industry to reduce the salt content of some foods [61]. However, effective public communication is also required to help populations reduce their sodium intake levels [62]. As indicated by the WHO in the guide SHAKE designed to help to implement strategies to reduce salt consumption in populations [63], it is important to implement integrated education and communication messages with other public strategies to raise public awareness of health risks and sources of salt in the diet to change behaviors. For parents, strategies could be implemented to improve their salt-related behaviors, including label checking, the use of iodized salt instead of regular salt/non-fortified salt [64], the promotion of a decrease in the use of table salt, and the improvement in the choice of foods with lower salt content. Future studies have an opportunity to determine the attitudes and parents' knowledge or motivation to change salt behaviors and then launch salt campaigns that aim to change salt-related behaviors in schoolchildren and parents. Additional information that is required includes whether parents and children know which foods have hidden salt, whether the salt content of the food has actually been reduced, and whether the population will accept foods with lower salt content.

This study has some limitations. First, because it is a cross-sectional study, cause–effect relationships cannot be established. Furthermore, the study sample is not a representative sample of the Spanish population because not all regions of Spain are represented. If we want to generalize our findings, we should do so with caution. However, it should be noted that a large sample was used, and it comprised subjects with a diverse socioeconomic status and was representative of several regions, including both rural/semi-urban and urban areas, in Spain. In addition, the study results may be subject to selection bias; from the total number of participants invited to participate in the study, only a small number finally entered. On the other hand, a 24-hour urine collection per participant is subject to high intra-individual and inter-individual variability [65]. However, single 24-hour urine collection per individual can be used to characterize differences in group mean intake and is not subject to systematic error when it is completed. In addition, 24-hour urine creatinine excretion was used as a marker to ensure sample completeness and quality [66]. The questionnaire that was used to determine the behaviors of parents and children was not a validated questionnaire because there is no one available for this population. However, some of the questions have been used in other studies [33,43,45,67–70], allowing their comparison with those in previous research. On the other hand, it is essential to bear in mind that the responses of the participants may be biased toward responses that they considered to be favorable. Among the strengths of our study is (1) the constancy of the significance in most of the factors in the unadjusted and UNa-24h-adjusted models, indicating that the findings are quite solid; (2) the use of an objective measure of sodium intake; and (3) the large sample size.

## Conclusions

The current levels of sodium intake are high in Spanish schoolchildren, and they have been associated with some parents’ salt-related behaviors. The use of iodized salt and table salt by the parents and children and the presence of a salt shaker on the table were associated with children’s sodium intake. It is important to make parents aware of the relationship between their behaviors regarding the use of discretionary salt and their children's sodium intake. Additionally, the low proportion of parents who looked at the sodium content on the label and the lack of a relationship between this practice and children’s sodium intake highlight a need for greater awareness and practical guidance to interpreting food labels. Educational messages, including reading food labels to select foods reduced in sodium, replace cooking salt using herbs or spices to flavor food, or avoid adding extra table salt will be useful to promote behavioral skills in parents and children related to salt intake.

## Supporting information

S1 Fig Adding table salt by the father and the mother. (PDF)

S1 File Questionnaire about behaviors related to salt. Spanish and English versions.
(PDF)
