# Supplementary figures and images for: The association of parents’ behaviors related to salt with 24 h urinary sodium excretion of their children: A Spanish cross-sectional study
Source: PLoS One. 2019 Dec 27;14(12):e0227035. doi: 10.1371/journal.pone.0227035 (PMC6934279; doi:10.1371/journal.pone.0227035)

**S1 Fig. Adding table salt by the father and the mother.**

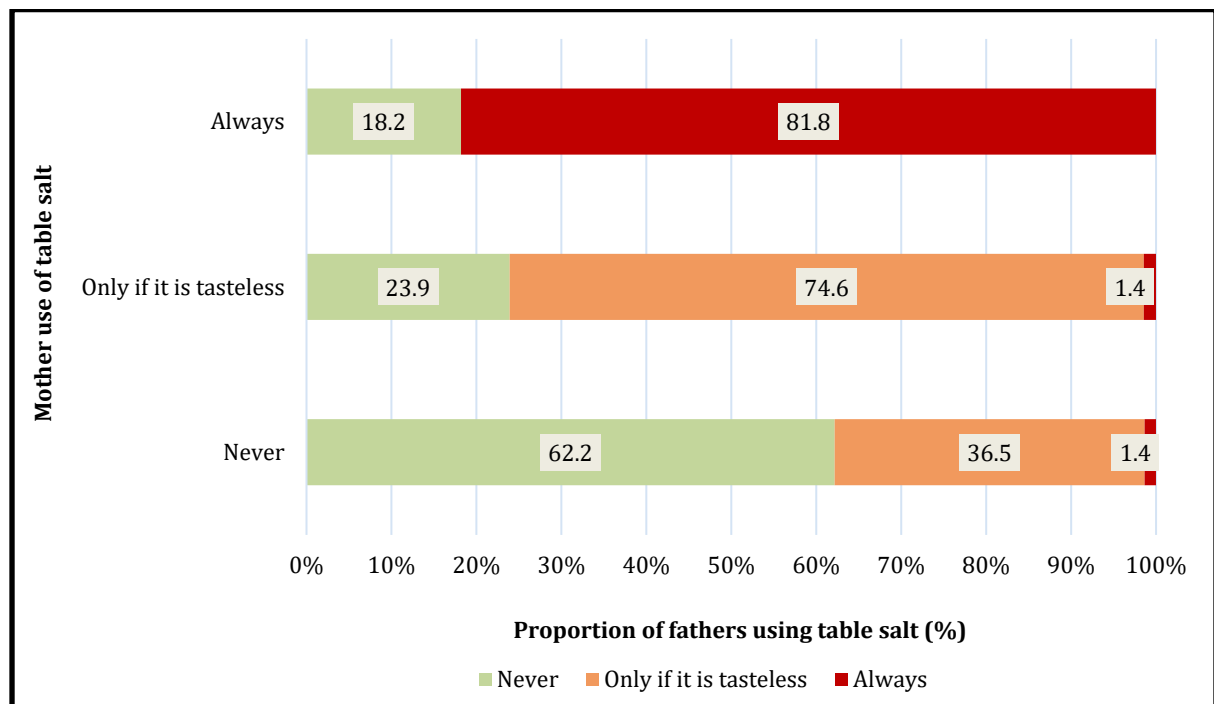

Supplement: S1 Fig — (PDF) [file pone.0227035.s001.pdf]
