# Supplementary material for: The association of parents’ behaviors related to salt with 24 h urinary sodium excretion of their children: A Spanish cross-sectional study
Source: PLoS One. 2019 Dec 27;14(12):e0227035. doi: 10.1371/journal.pone.0227035 (PMC6934279; doi:10.1371/journal.pone.0227035)
Supplement: S1 File — Spanish and English versions. (PDF) [file pone.0227035.s002.pdf]

UNIVERSIDAD COMPLUTENSE DE MADRID
DEPARTAMENTO DE NUTRICION

Facultad de Farmacia
Ciudad Universitaria
28040 Madrid
Tel: +34 91 394 18 10
Fax: +34 91 394 17 32

CUESTIONARIO DE HÁBITOS SOBRE LA SAL
(A rellenar por los progenitores/madres/tutores)

Nombre y apellidos del niño/a:
____

1. *En su hogar ¿añaden sal a los alimentos mientras se cocina?*
   Si ◻
   No ◻

2. *¿Añaden sal a los alimentos al consumirlos y después de haber sido cocinados?:*

PADRE		***MADRE***	
Sí, siempre, antes de probarlos		*Sí, siempre, antes de probarlos*	
Sólo si están sosos		*Sólo si están sosos*	
No, nunca añado		*No, nunca añado*	

3. *En su hogar ¿utilizan sal yodada o sal convencional?*
   Sal yodada ◻
   Sal convencional ◻
   Otros

4. *En su hogar ¿consultan habitualmente el etiquetado de los alimentos para conocer su contenido en sal?*
   Si ◻
   No ◻
   A veces ◻

5. *En su hogar, ¿el salero está en su mesa a disposición de quien lo desee?*
   Si ◻
   No ◻
   A veces ◻

6. *A su hijo/a, ¿le gustan los alimentos salados/sosos?*
   Salados ◻
   Sin sal ◻
   Contenido medio de sal ◻

UNIVERSIDAD COMPLUTENSE DE MADRID
DEPARTAMENTO DE NUTRICION

Facultad de Farmacia
Ciudad Universitaria
28040 Madrid
Tel: +34 91 394 18 10
Fax: +34 91 394 17 32

7. *Indique ¿Con qué frecuencia se añade su hijo sal en la mesa?*

	**Más de 1 vez día**	**1 vez día**	**4-6 veces semana**	**2-3 veces semana**	**1 vez semana**	**1-3 veces mes**	**Nunca**
Sal añadida en la mesa							

UNIVERSIDAD COMPLUTENSE DE MADRID
DEPARTAMENTO DE NUTRICION

Facultad de Farmacia
Ciudad Universitaria
28040 Madrid
Tel: +34 91 394 18 10
Fax: +34 91 394 17 32

(ENGLISH VERSION)

SALT HABITS QUESTIONNAIRE
(to be completed by parents/guardians)

Name and surname of the child:

____

1. *In your home, do you add salt to the food while cooking?*
   Yes ◻
   No ◻

2. *Do you add salt to food when you eat it after it is cooked?*

FATHER		***MOTHER***	
Always		*Always*	
Only if it is tasteless		*Only if it is tasteless*	
Never		*Never*	

3. *In your home, do you use iodized salt or regular salt?*
   Iodized salt ◻
   Regular salt ◻
   Others ◻

4. *In your home, do you routinely check food labels for salt content?*
   Yes ◻
   No ◻
   Sometimes ◻

5. *In your home, is the salt shaker on your table for anyone who wants it?*
   Yes ◻
   No ◻
   Sometimes ◻

6. *Does your child prefer salty foods or not salty?*
   Very salty ◻
   Not salty ◻
   Somewhat salty ◻

UNIVERSIDAD COMPLUTENSE DE MADRID
DEPARTAMENTO DE NUTRICION

Facultad de Farmacia
Ciudad Universitaria
28040 Madrid
Tel: +34 91 394 18 10
Fax: +34 91 394 17 32

7. *How often does your child add salt to the food after it is cooked?*

	**More than one day**	**One time per day**	**4-6 times per week**	**2-3 times per week**	**Once a week**	**1-3 times per month**	**Never**
Use of table salt							
